# Antibacterial Properties of Polymeric Membranes Containing Doxycycline for Potential Applications in Foot Ulcer Treatment

**DOI:** 10.3390/ijms26073274

**Published:** 2025-04-01

**Authors:** Stevaly Pérez-Gutiérrez, Jesús Ángel Ramírez-Enciso, Laura Abisai Pazos-Rojas, Abigailt Flores-Ledesma, Eric Reyes-Cervantes, Diana del C. Pazos-Guarneros, Ismael Juárez-Díaz, Paola G. Gordillo-Guerra, Bernardino Isaac Cerda-Cristerna, José Luis Suárez-Franco, Carolina Samano-Valencia, Brenda Erendida Castillo-Silva, Alejandro G. Martínez-Guerrero, Gisela N. Rubin de Celis-Quintana, Alberto V. Jerezano-Domínguez

**Affiliations:** 1 Faculty of Medicine, Meritorious Autonomous University of Puebla (BUAP), Puebla 72410, Mexico; stevaly.perezg@alumno.buap.mx (S.P.-G.); jesus.ramirezen@alumno.buap.mx (J.Á.R.-E.); 2 Faculty of Stomatology, Meritorious Autonomous University of Puebla (BUAP), Puebla 72410, Mexico; abigailt.flores@correo.buap.mx (A.F.-L.); diana.pazosg@correo.buap.mx (D.d.C.P.-G.); ismael.juarez@correo.buap.mx (I.J.-D.); carolina.samano@correo.buap.mx (C.S.-V.); brenda.castillosilva@correo.buap.mx (B.E.C.-S.); alejandro.mguerrero@correo.buap.mx (A.G.M.-G.); gisela.rubin@correo.buap.mx (G.N.R.d.C.-Q.); 3 Tecnológico de Monterrey, Escuela de Ingeniería y Ciencias, Puebla 72453, Mexico; 4 Direccion de Innovación y Transferencia de Conocimiento, Meritorious Autonomous University of Puebla, Puebla 72592, Mexico; eric.cervantes@correo.buap.mx; 5 Departamento de Sistemas Biológicos, Unidad Xochimilco, Universidad Autónoma Metropolitana, Coyoacan, Calzada del Hueso 1100, Col. Villa-Quietud, Ciudad de México 09310, Mexico; pgordillo@correo.xoc.uam.mx; 6 Facultad de Odontología, Universidad Veracruzana, Región Orizaba-Córdoba, Orizaba 94732, Mexico; bcerda@uv.mx (B.I.C.-C.); jsuarez@uv.mx (J.L.S.-F.)

**Keywords:** chitosan, carboxymethylcellulose, membrane, foot ulcers *Escherichia coli*, *Staphylococcus aureus*, *Streptococcus mutans*

## Abstract

Membranes made from biopolymers and loaded with doxycycline were investigated for potential use in the treatment of foot ulcers in diabetic patients. Carboxymethylcellulose (CMC) and chitosan (CHS) membranes were fabricated with 7% glycerol and 1% doxycycline (DOX). Their mechanical and physical properties, biocompatibility, and antimicrobial effects were thoroughly evaluated. The results demonstrated effective antibacterial activity against *S. aureus* and *S. mutans*. Based on the mechanical, physical, and hemolytic data, DOX-loaded CMC/CHS/G membranes show promise as a topical wound delivery system.

## 1. Introduction

Infection of the foot is a common complication in patients with diabetes mellitus, and it can lead to significant morbidity and mortality. Healthcare interventions using antimicrobial therapy can effectively reduce morbidity, mortality, and associated economic costs [1,2,3]. The addition of a 2% doxycycline hyclate (DOX) solution applied locally to the standard care regimen for chronic foot ulcers has been reported to have a positive effect, particularly in reducing wound size during the first two weeks and promoting healing until complete closure [4]. Membranes as transdermal patches are a convenient and non-invasive method of drug delivery. They offer a sustained release of antibiotics, maintaining constant drug levels in the topical area. This treatment improves patient compliance due to its ease of use and long-lasting effects [5] making it easier for patients to adhere to the regimen. In addition, this topical healthcare may improve the tissue.

New membranes based on carboxymethylcellulose/chitosan/glycerol can be used to deliver doxycycline (DOX) for the topical therapy of diabetic foot. DOX is a synthetic antibiotic derived from the tetracycline family; it is a bacteriostatic agent used to treat Gram-negative and Gram-positive bacteria [6], including *Staphylococcus aureus* and *Escherichia coli* [7]. Carboxymethylcellulose (CMC), a derivative of cellulose, is widely used in the manufacture of advanced dressing and drug delivery systems due to its many physicochemical properties, non-toxicity, high chemical stability, suitable biodegradability, and the potential for local drug release on tissue [6,8]. Chitosan (CHS) is a versatile natural copolymer derived from chitin with mucoadhesive properties, biocompatibility, biodegradability, and antimicrobial activity for the treatment of chronic and infectious wound healing [9]. Loading glycerol (G) into CHS-based membranes can be used to design a drug delivery system with the appropriate mechanical and antibacterial properties [10] to maintain intimate contact with the local tissue.

Considering the properties of doxycycline (DOX) and polymers used to treat diabetic foot ulcers, in this study we proposed to fabricate a DOX-loaded membrane based on these biopolymers. In this study, membranes of CMC, CHS, and G containing DOX were evaluated for their physio-chemical characteristics and antibacterial activity against *S. aureus*, *E. coli*, and *S. mutans*. This treatment may offer a new alternative, with an improved efficacy, in the management chronic foot ulcers, promoting wound healing and improving patient compliance.

## 2. Results and Discussion

### 2.1. Physical Appearance and Scanning Electron Microscopy Analysis

**Figure 1:**
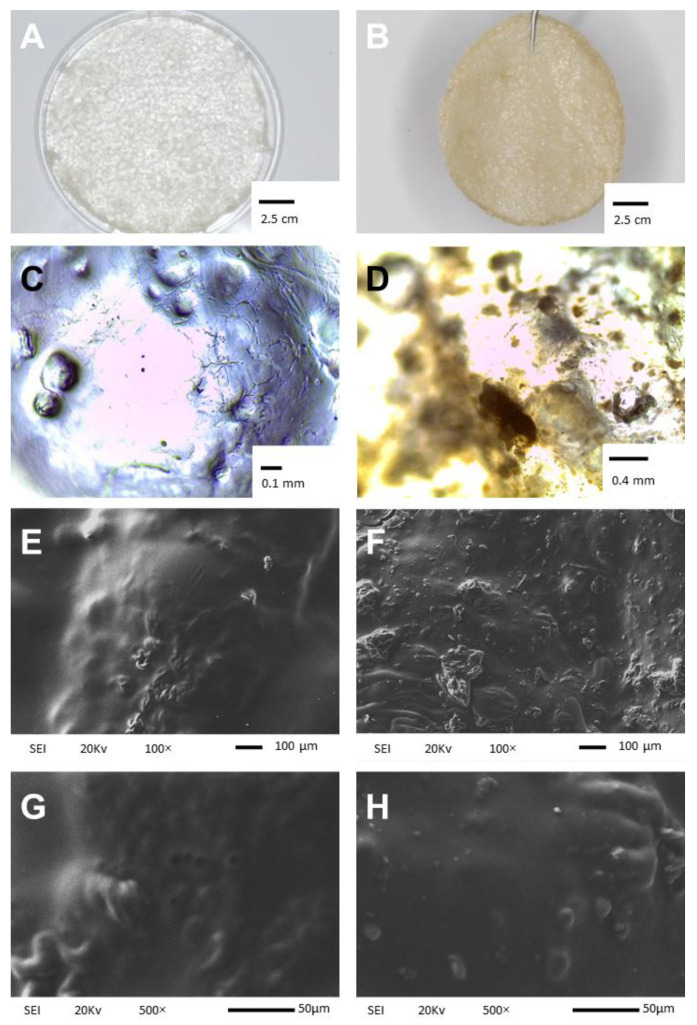
Physical appearance of MControl (**A**) and MDOX (**B**) membranes; optical images of the surface of MControl (**C**) at 4× and MDOX (**D**) at 10× after 5 min in PBS; scanning electron microscope images of the surface of MControl at 100× (**E**) and 500× (**G**) magnifications. Scanning electron microscope images of the surface of MDOX at 100× (**F**) and 500× (**H**) magnifications.

Similarly to CHS, CMC is a water-soluble anionic biopolymer with excellent physio-chemical properties, such as hydrophilicity, non-toxicity, and bio-adhesiveness [11]. Figure 1 shows images at three levels. Section A and B shown photographs taken with the HONOR X8b, Model LLY-LX3 where it can be observed that G (MControl) and Doxycycline-G (MDOX) were homogeneously integrated into the membrane (Figure 1A,B, respectively). In the microphotographs taken with a Leica CC50 camera, coupled to a Leica DM1000 optical microscope, MDOX presents a brown color (Figure 1D) due to the presence of the antibiotic, similarly to the study by Dinte et al. [12]. In contrast, MControl is colorless (Figure 1C), as observed in biopolymer membranes reported by other authors [10,13,14]. Finally, in Sections 1E, 1F, 1G, and 1H, the top surface of MControl and MDOX was examined by SEM after incubating at 37 °C for 72 h to investigate the influence of DOX on the membrane morphology. Surface morphology analysis is a widely used technique to study the sub-microscopic details of different drug delivery systems in biopolymeric matrices. Figure 1E shows the top surface of a CMC/CHS/G membrane, where few agglomerations of CMC with CHS can be observed in the matrix resulting from the synthesis method. The SEM images at 100× (Figure 1F) show the surface morphology of the CMC/CHS/G membranes with DOX at 1%, suggesting that the prepared membranes loaded with DOX had an irregular surface, in contrast to the unloaded membranes (Figure 1E). DOX particles were visible in the form of dots (Figure 1H) over the membrane, but were absent from the rough surface of MControl (Figure 1G); similar images were reported by Iqbal et al. [15]. The DOX-loaded hydrogel membrane prepared by Ulu et al. [11] also exhibited a similar morphology. This compact surface may be suitable for wound applications due to its non-porous structure, which inhibits the penetration of bacteria or liquids into the membrane [16].

### 2.2. FTIR−ATR Analysis

**Figure 2:**
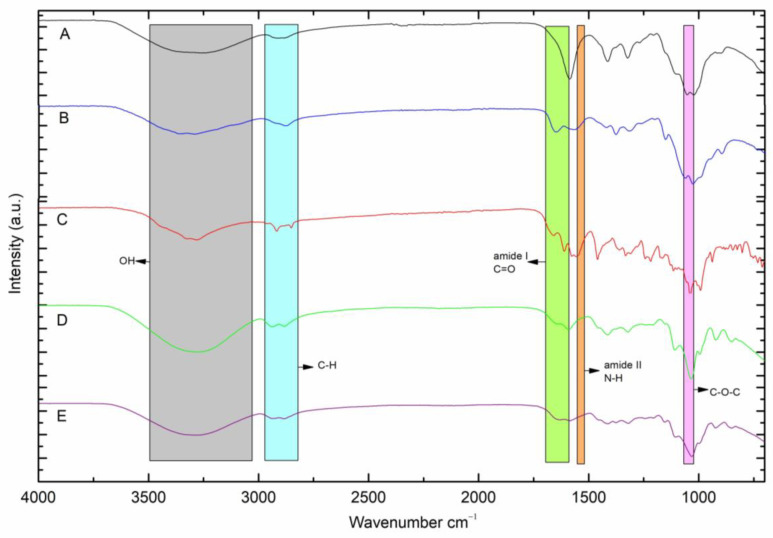
FTIR-ATR spectra of (**A**) solid CMC, (**B**) solid CHS, (**C**) DOX, (**D**) MControl (**E**) MDOX.

To analyze the interaction between the DOX functional groups and the CMC/CHS/G membrane, the FTIR spectra of the CMC/CHS/G membrane and the CMC/CHS/G/DOX membrane were characterized (Figure 2D,E). The FTIR spectrum of pure DOX exhibited characteristic absorption peaks between 3000 and 3500 cm^−1^ (OH and NH), 1610 cm^−1^ (amide band I), and 1576 cm^−1^ (amide band II) (Figure 2C), similarly to those reported by other authors [6,11,17]. The characteristic IR bands of the powdered CMC show the O−H and stretching vibration of the COO-bond at 3315 cm^−1^, while powdered CHS−FTIR shows bands at 3287 cm^−1^ corresponding to the stretching vibration of N−H. Additionally, peaks at 2872 and 2280 cm^−1^ are assigned to the typical C−H stretching vibration in −CH_2_ and −CH_3_ of CHS, respectively. In addition, the peak at 1648 cm^−1^ corresponds to C=O stretching (amide I). The peak at 1375 cm^−1^ is assigned to the C−N stretching bond (amide II), and the peak at 1025 cm^−1^ is assigned to the −OH bond of CHS. When DOX was added to the CMC/CHS/G membrane, the peak shifted from 3253 cm^−1^ (Figure 2D) to 3276 cm^−1^ (Figure 2E), which indicates the N−H and the O−H bond. The weak stretching vibration bonds at 2883.52 and 2935.59 cm^−1^ correspond to the asymmetric C−H groups in MControl and MDOX. In the spectra of the DOX-loaded CMC/CHS/G membrane (Figure 2E), characteristic peaks in the range of 1583.52 cm^−1^ indicated the presence of DOX in the CMC/CHS membrane, similar as reported in the literature [13,15,17]. The peaks at 1413.79 and 1031.89 cm^−1^ in the CMC/CHS/G membrane (Figure 2D) and 1408.00 and 1028.03 cm^−1^ in the CMC/CHS/G at 1% with DOX (Figure 2E) appeared to belong to the C−O and C−O−C groups, respectively.

### 2.3. Tensile Properties

The mechanical properties of the membranes are critical for their successful application in tissues such as human skin. Mechanical compatibility between the membrane and skin tissues is required to mimic the extracellular matrix. For this reason, it is desirable for the biopolymeric matrix to have mechanical properties similar to those of human skin, providing good elasticity and adaptability to movement [14]. In the literature, the tensile strength of the base linear stiffness of a hydrated acellular collagen scaffold was determined to be 0.023 ± 0.04 MPa but increased significantly to 0.072 ± 0.021 MPa when fibroblasts and keratinocytes remodeled the matrix for two weeks [18]. Additionally, the elastic modulus is a crucial mechanical cue for cells [19,20]. The results of mechanical stability testing, including tensile strength and elongation at break values, are listed in Table 1. It was seen that the addition of DOX resulted in tensile strength values similar to those of MControl and proportional to the elongation values, 0.07 Mpa and 0.09 Mpa, respectively. The maintenance of mechanical properties can be attributed to the low doses of DOX in the biopolymeric matrix of the membranes, demonstrating that these values are comparable to those of the skin.

### 2.4. Mass, Thickness and Roughness of the Membrane

The mechanical properties of the membranes, such as mass, thickness, and roughness, also provide insights into the membrane structure. The membranes are three-dimensional networks formed by cross-linking monomers or polymers with good mechanical strength, biodegradability, and biocompatibility [21]. The mass values of membranes, as shown in Table 1, demonstrated an increase in the mass of MDOX compared to MControl (9.251 ± 0.688 mg and 6.051 ± 0.306 mg, respectively). The difference in membrane mass values was statistically significant (*p* < 0.001). The observed increase in mass values is due to DOX loading in the CMC/CHS/G membrane. The properties of the membranes demonstrate a high potential for use in the manufacture of dressings, with ability to be used to fill spaces, function as wound dressings, or serve as drug delivery systems [22]. In practical situations, the adaptability of membranes can fill irregular spaces from the wound [23]. In this sense, the thickness values were of 604 ± 0.182 µm and 833 ± 0.134 µm for MControl and MDOX, respectively (Table 1), with a statistically significant difference (*p* < 0.005). These values are comparable to the measure thickness of grafted skin substitutes (763 ± 80 µm) and autografts (761 ± 74 µm) [18]. This change in membrane thickness was caused by the adsorption of DOX by CMC and CHS, as suggested by Tang et al. [13]. The increase in thickness obtained in MDOX is due to ionization states of doxycycline [24] and its interaction with polymers, forming hydrogen bonds within the drug-delivery system [25]. The rough surface of the membrane may also enhance tissue and promote cellular activity [26]. The incorporation of CMC and CHS in combination with G further induced a skin adhesive character in the membranes. The cross-linking of these biomaterials results in the formation of an interpenetrating biopolymeric network, creating a rapid method for producing a drug delivery system with the necessary thickness, mass, and roughness properties [27].

### 2.5. Surface pH Kinetic

**Figure 3:**
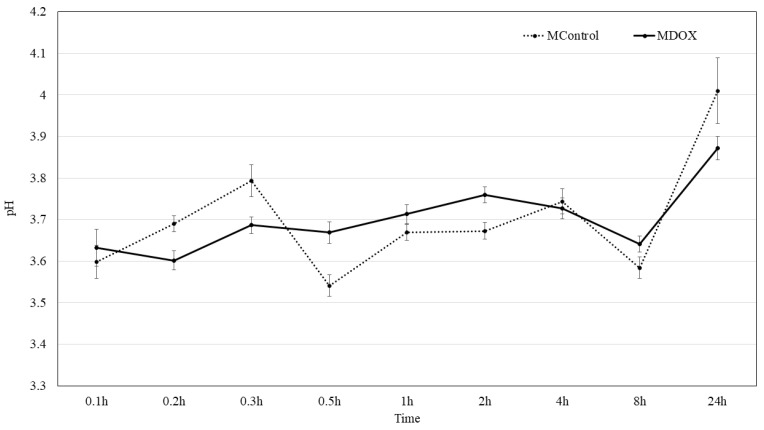
Graphical presentation of surface pH kinetic of MControl and MDOX.

DOX existed in the form of DOX^+^ at pH 2–3, while a high concentration of H^+^ ions competes with DOX^+^ on the membrane surface, influencing the adsorption performance. The absorption efficiency of DOX in the biopolymeric matrix of the membranes is influenced by pH changes, as reported Tang et al. [13]. The pH of the surface membrane surface was 3.59 and 3.79 in the buffer solution for MControl and MDOX, respectively. Then, slowly increasing over time, a gradual increase in solution pH from 3.6 to 4.1 suggests the release of DOX from the biopolymeric matrix, as also indicated by Tang et al. [13]. However, drug release tests are necessary to fully evaluate DOX release from membranes. The pH range 3–4 observed here differs from that reported by Dinte et al. [12], who suggested that a pH range of 5.5–7.0 is well tolerated for skin adhesive film. Nevertheless, at a local pH below 4 for up to 8 h, as shown in Figure 3, bacteria do not survive. Additionally, at a pH above 2.8 the fibroblast remain viable, allowing them to utilize the local anion gap to proliferate and promote healing in a bacteria-free environment [28].

### 2.6. Moisture Sorption Water Sorption Behavior and Swelling Capacity

**Figure 4:**
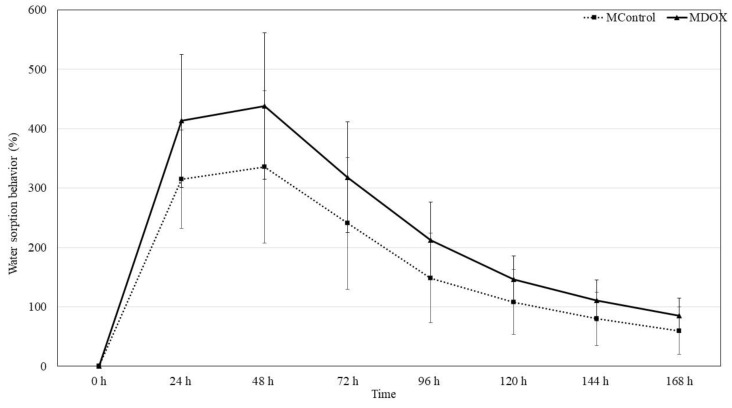
Graphical presentation of water sorption behavior of MControl and MDOX.

CMC and CHS are commonly used as drug delivery systems due to their strong hygroscopicity. Moisture sorption, water sorption behavior, and swelling properties of natural materials depend on the nature of the medium and on the swelling caused by solvent diffusion into the material structure from the extracellular medium. The stability of DOX could be affected by membrane moisture sorption during storage, as it is a humidity sensitive drug [29]. In addition to their capacity to swell and absorb wound exudate, these biopolymer membranes maintain wound moisture by delivering water molecules [22]. Table 1 shows the moisture sorption weight gain recorded on the tenth day. The moisture sorption of these membranes follows the order of MControl < MDOX, with values of 37.52 ± 6.54% and 205.01 ± 66.94%, respectively. The difference is statistically significant (*p* < 0.002). Similar results were reported by Peng et al. [29]. The introduction from charged chemical groups from doxycycline into the biopolymeric matrix can alter some membrane characteristics. Therefore, the impact of these charges on moisture sorption and water sorption behavior were evaluated (Figure 4). The loading-DOX membrane exhibited a higher moisture sorption (Table 1). The process of hydration in polymer networks can absorb at least 10% of their dry weight in water and in some cases even thousands of times that amount [27]. The water sorption behavior was analyzed at 24, 48, 72, 96, 120, 144, and 168 h. The highest water sorption in both membranes was observed at 48th hour in PBS (pH = 7.4, 25 °C), with values 335.71 ± 128.51% and 437.81 ± 123.32% for MControl and MDOX, respectively (Table 1). The DOX-loaded membrane exhibited higher moisture sorption (Table 1). This increase was due to the incorporation of DOX into the biopolymeric matrix, likely through the interaction of acid groups (-OH, -CONH_2_, NMe_2_), which led to a greater water affinity [26]. Due to their high water absorption capacity and biocompatibility, these membranes have been used in dressing and drug delivery as transdermal systems [30] and in preventing the wound from drying out in diabetic foot ulcers [22,31]. These membranes are cross-linked polymer networks that swell in a liquid medium. The embedded liquid acts as a selective filter, allowing the free diffusion of certain solute molecules, such as drugs, while the polymer network serves as a matrix to retain the liquid [24]. The swelling behavior of biopolymeric-based membranes was tested in buffer media. The swelling index was studied to estimate the swelling behavior of the membranes in contact with wound fluids after the application to the ulcer. Results indicated that membranes showed similar swelling capacity to MControl and MDOX, 0.52 ± 0.05 and 0.53 ± 0.03, respectively (Table 1). This behavior can be explained by the fact that CMC/CHS/G is a good hydro dispersible biopolymer base that allows for the faster swelling of DOX, similar to the results reported with other membranes loaded with DOX [12]. These results indicate that 100 mg was the ideal amount of the drug incorporated into a 17.5 mg/mL CMC and 8.75 mg/mL of CHS powder polymer blended membrane.

### 2.7. Disintegration or Biodegradability

**Figure 5:**
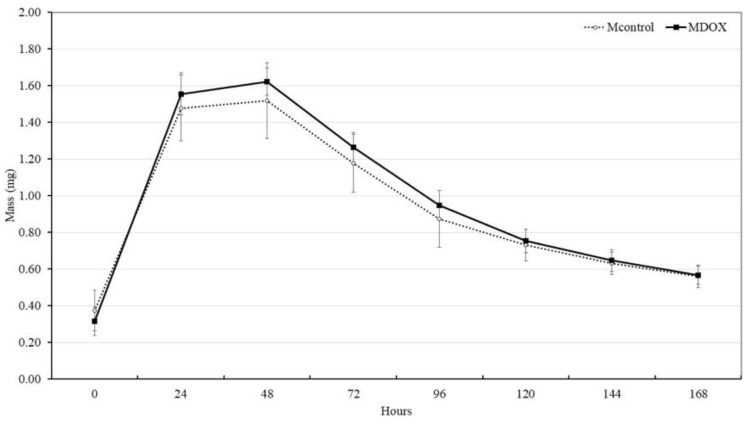
Graphical presentation of MControl and MDOX disintegration.

The biodegradation potential of the membrane is important for the formation of the extracellular matrix and skin repair. In tissue engineering, the scaffold should not be completely removed until the wound healing is complete. To evaluate the biodegradability potential of the CMC/CHS/G membrane, an SBF solution, which closely resembles human blood plasma, was used. The degradation behavior of the membrane was investigated over time (0, 1, 2, 3, 4, 5, 6, and 7 days) in the SBF solution at 37 °C and the results are shown in Figure 5. All of the membranes did not degrade after 1 week and maintained their weight during the second week (Figure 5). The mass gain values of MDOX and MControl exhibited similar behavior at 1, 2, 3, 4, 5, 6, 7, and 8 days but showed a statistically significant difference at time 0 h (*p* < 0.05). The membranes were formed through hydrogen bonds, ionic interactions, and other non-covalent interactions. These bonds maintained their structural integrity, forming a viscoelastic biomaterial with implantable ability, self-healing properties, and superabsorbency for wound tissue remodeling [9].

### 2.8. Blood Coagulation

The PT test and the APTT evaluated the effect of the membranes on the extrinsic and intrinsic pathways of blood coagulation, respectively. Table 1 shows the PT and APTT results for CMC/CHS/G and CMC/CHS/G/DOX. The unloaded membranes induced a PT and APTT within the normal range. Similarly, the DOX-loaded membranes also induced a PT and APTT within the normal range. These findings showed that the membranes did not interact with the blood coagulation pathways. Although membranes composed of CMC and CHS have demonstrated hemostatic properties, their hemostatic effect depends on both the membrane composition and the percentage of these polymers [32,33]. In this study, the samples were prepared with a percentage of CMC and CHS that did not induce any effect on the PT and APPT. Additionally, doxycycline is an antibiotic with various biological effects; however, these do not include the induction or inhibition of blood coagulation [34].

### 2.9. Hemolytic Properties

The design of the biomaterials intended for wound care requires careful consideration of their interaction with biological systems. Given that the fabricated membranes are intended to come into direct contact with diabetic foot wounds, ensuring their blood compatibility is a critical requirement to prevent adverse reactions and promote healing. The CMC/CHS-based membranes, widely recognized for their hemocompatibility, are generally well tolerated by the human body and do not induce an immune response or inflammation [35]. The hemolytic properties of the membranes were evaluated by quantifying the extent of hemolysis induced by the CMC/CHS membrane (MControl = 16.85%) and the Doxycycline-loading membrane (MDOX = 14.72%). According to the ASTM standard recommendation followed in the experiment, the two membranes showed hemolytic properties (>5% of hemolysis), but the DOX-loaded membrane induced a hemolytic level lower than the unloaded membrane. The CMC/CHS/G-based membrane exhibited a hemolytic effect due to detergent action of the cationic charge in the biopolymeric matrix, which disrupts the red blood cells [36]. Interestingly, the DOX-loaded membranes at 1.08% induced a lower percentage of hemolysis, suggesting that the addition of the antibiotic might reduce the cationic charges. Indeed, polymer-based films improved their blood compatibility properties when DOX was added to the formulation [37].

### 2.10. Antibacterial Activity of CMC/CHS/G/DOX Membranes

The inhibition results for *Staphylococcus aureus*, *Escherichia coli*, and *Streptococcus mutans* using CMC/CHS/DOX membranes indicated that the conformation of these membranes does not significantly affect the inhibition capacity against the evaluated strains (Table 2). In both repetitions, a constant and effective inhibition of *S. aureus*, *E. coli*, and *S. mutans* growth was observed. Inhibition was complete for *S. aureus* and *S. mutans*, and partial for *E. coli*, where in both repetitions the bacterial count was lower than the initial inoculum. As observed, there is no significant difference in the inhibition capacity of the CMC-CHS-DOX membranes, suggesting that the proposed formulation allows for membrane synthesis without compromising the inhibition capacity of the active ingredient or antibiotic contained in them. It is important to note that carboxymethylcellulose, glycerol, and chitosan do not exhibit any inhibitory capacity against *S. mutans*, *S. aureus*, and *E. coli* (Table 2); the membranes without doxycycline allowed for the adequate growth of all evaluated strains. When conducting inhibition tests using the sensidisc method, an average inhibition halo of 25.5 mm was observed for all evaluated strains. Although the inhibition halo technique [38], and agar well diffusion [39] are commonly used to assess the ability of biomaterials to inhibit bacterial strains, counting bacteria in the presence of biomaterials provides more detailed information on their effective inhibition [40]. Our results indicate that the inhibition halo test did not provide information as detailed as the results presented in Table 2, where total inhibition of *S. mutans* and *S. aureus* was observed, similarly to previous studies [17,41]. Previous research has shown that the inhibitory effect of Doxycycline has a wide range of activity against Gram-positive and Gram-negative bacteria [41,42]. In contrast, the inhibition halos formed by the MDOX membranes showed only partial inhibition for all the evaluated strains.

Among the characteristics of doxycycline as an antibiotic is its systematic action in various tissues. Its high lipophilicity allows it to penetrate numerous membranes and reach target molecules. Tetracyclines act as cationic coordination complexes to cross OmpF and OmpC porins channels in Gram-negative bacteria. Similarly, in Gram-positive bacteria, the neutral, lipophilic form penetrates the cytoplasmic membrane. Passage through the cytoplasmic membrane is energy-dependent and driven by the proton-motive force [4]. The bacteriostatic action of DOX aims to inhibit bacterial growth by allosterically binding to the 30S prokaryotic ribosomal unit during protein synthesis. DOX prevents the binding of charged aminoacyl-tRNA (aa-tRNA) to the A-site of the ribosome, halting the elongation phase and leading to an unproductive cycle of protein synthesis. Doxycycline affects the binding rate of triple complex formation with the ribosome. The development of microbial resistance to antibiotics poses a potential risk. However, the local application of 1% doxycycline results in a significantly higher concentration (i.e., 10,000 mg/mL) than the minimum inhibitory concentration required for a 50% reduction in pathogenic growth, thereby minimizing the likelihood of developing doxycycline-resistant bacteria (Level IV) [4,43]. Considering the characteristics of this antibiotic and its ability to inhibit bacteria associated with diabetic ulcers, we propose CMC/CHS/G/DOX membranes as a release system with effective antibacterial properties. This system represents a promising alternative treatment to promote tissue recovery and restoration in patients affected by diabetic ulcers. Furthermore, when these membranes were tested for water absorption and swelling capacity in BPS, they demonstrated promising results for their potential use in skin. An additional advantage is their ability to be applied directly to the infection site, enhancing their therapeutic effectiveness.

## 3. Material and Methods

### 3.1. Formulation and Synthesis of Membranes

Carboxymethylcellulose sodium (CMC, with a molecular weight of 90 kDa and a degree of substitution of 0.7) and high molecular weight (CHS) in powder form, with a deacetylation degree greater than 85%, were obtained from Sigma-Aldrich (Saint Louis, MO, USA). Glacial acetic acid and distilled water were purchased from Sigma Aldrich (USA). Glycerol spectrophotometric grade (99.5%+), was obtained from Acros, NJ, USA. Doxycycline was purchased from RAAM De SAHUAYO Lab. S.A. DE C.V. Reagents for prothrombin time (PT) and activated partial thromboplastin time (APTT) were obtained from Spinreact (Estado de México, MEX). The hemolysis test was performed using cyanmethemoglobin reagent (Hycel Reactivos Químico, Jalisco, MEX) and standard hemoglobin was acquired from Spinreact.

#### Preparation of CMC/CHS Membrane

The membranes were prepared using a modified casting and solvent evaporation method as reported by Hachity et al. [10]. To prepare 17.5 mg/mL of CMC and 8.75 mg/mL of CHS powder solution, 40 mL of deionized water was added to a flask and magnetically stirred (400 rpm) at 60 °C. An aqueous solution of acetic acid (2%, *v*/*v*) was then added to the mixture, and stirring continued for 15 min until the CMC and CHS were completely dissolved, resulting in a CMC/CHS solution. Simultaneously, glycerol (G) was added at concentration of 7% (*v*/*v*), and stirring continued for 15 min until the CMC/CHS/G solution transformed into a flocculent suspension. Forty mL of the CHS suspension, were poured into Petri dishes (16 cm in diameter) on an analytical scale. The prepared plates were dried at room temperature for 72 h until the water had completely evaporated. The final sample designation and amounts of CMC, CHS, G and DOX used for each sample were summarized in Table 3. The MDOX was prepared with the same formulation, using 100 mg of doxycycline hyclate (DOX) to produce a membrane containing 1.08% of DOX.

### 3.2. In Vitro Characterization of the Membranes

#### 3.2.1. Visual Inspection and Evaluation of Content Uniformity in the Membranes

The samples were analyzed with an optical microscope Leica DM-1000 using a Leica CC50 camera at 4× and 10× magnifications to identify the presence of any imperfections, both after preparation and during storage. The uniformity of the membranes was assessed by cutting disks with a diameter of 0.6 cm from five different areas of the studied TP samples.

#### 3.2.2. Scanning Electron Microscopy Analysis

The surface images of the TP samples were observed using a scanning electron microscope (SEM, JEOL, JSM-6610 LV, Tokyo, Japan). The membranes were dried at 75 °C for 48 h, after which the dried fragments were broken and coated with gold by sputtering to produce electric conductivity. SEM images of the membrane surface were captured under low vacuum condition operating at 5 kV.

#### 3.2.3. Fourier Transform Infrared (FTIR-ATR) Spectra Analysis

The spectra of the CMC/CHS samples and their DOX sample were obtained using Fourier transform infrared spectroscopy (FTIR-ATR) with (attenuated total reflectance/ATR) an accessory (Bruker, Vertex 70 model, Bulington, ON, Canada), with 32 scans in the range between 400 and 4000 cm^−1^, and a resolution of 4 cm^−1^.

#### 3.2.4. Tensile Strength and Elongation

The tensile tests were performed according to the method described in the literature [20,44] using a universal testing machine (Instron 4465, Instron, Norwood, MA, USA) at 5 mm/min crosshead speed. All samples were cut to the standard shape of 20 mm wide and 150 mm gauge length. The measurement was performed in an ambient condition (25 °C, relative humidity of 48 ± 2%). Tensile strength and elongation at break were evaluated (*n* = 10), and the average values were reported for accuracy.

#### 3.2.5. Mass and Thickness of Membranes

The samples were weighed separately using the analytical balance (OHAUS Adventure Pro AV265C, New Jersey, EE.UU.: 260g (SD ± 0.1 mg) (l ± 0.3 mg), after 48 h of being stored at room temperature. The mean mass and standard deviation (SD) were calculated. The thickness of the membrane was measured using a micrometer screw (SHAHE-Model T152002FR, Wenzhou, China, 0–25 ± 0.04 mm) in the center and each corner, taking an average of the ten values per unit (*n* = 10) [10].

#### 3.2.6. Roughness

The membrane surface roughness parameters were measured with a previous roughness meter (Mitutoyo SJ-301, Mitutoyo America Corporation, Kanagawa, Japan), working at a speed of 0.25 mm/s and a cutoff distance of 0.8 × 5 mm [45]. The membranes were fixed with double-sided tape parallel to the tip of the roughness gauge. Five samples were used (*n* = 10).

#### 3.2.7. Surface pH

A disk unit (*n* = 10) was placed in a flask containing 5 mL of deionized water. The formulation was allowed to swell for 5 min and was subsequently removed from the flask [46]. The resulting pH of the liquid was recorded using a pH meter (ROCA-Model PHS-3CU) at 25 °C and was taken as an indication of the formulations surface pH.

#### 3.2.8. Moisture Sorption

The membrane’s moisture sorption was studied by exposing them to 75% relative humidity (RH) using a desiccator with an oversaturated NaCl solution at room temperature. The units were pre-weighed (m1) and stored in a humid desiccator for 10 days (*n* = 10). Afterwards, they were re-weighed (m2). Moisture sorption was calculated in the same manner as swelling capacity and multiplied by 100 to obtain the sorption percentage [46]. The experiment was performed with ten samples.

#### 3.2.9. Water Sorption Behavior

CMC/CHS membranes and DOX loading membranes were cut into small disks (0.6 cm diameter, *n* = 10), desiccated overnight under vacuum, and weighed to determine their dry mass. The weighed disks were placed in a flask containing 5 mL of phosphate-buffered saline (PBS, pH 7.4) at 25 °C [11]. The swelling kinetics were evaluated by periodically measuring the weight increment of the samples with respect to the dry membranes, using an analytical scale with a precision of 0.001 g. After gently bottling the surface with a tissue to remove the excess PBS solution, the samples were weighed, and the respective values were recorded until day 7.

The water sorption (W.S.) was calculated as follows:

(1) $$W.S.\;\left(\%\right)=\frac{m2-m1}{m1}\times 100\;$$

where m1 expressed the weight of the dried membranes, while m2 expressed the weight of the swollen membranes.

#### 3.2.10. Swelling Capacity

The swelling capacity of the membranes was evaluated using a modified test from the literature [46,47], where a membrane with a determined mass (m1) was placed in a glass flask and immersed in 5 mL of PBS (pH = 7.38) at 37 °C. The membrane was allowed to swell for 5 min. Its mass (m2) was recorded after gently wiping the product with a piece of tissue paper to remove the surface water. The swelling index represents the mass gained with respect to the mass of a dehydrated membrane and was calculated according to Equation (2). The experiment was carried out ten times.

(2) $$S.I.=\frac{m2-m1}{m1}$$

#### 3.2.11. Evaluation of Disintegration or Biodegradability

The disintegration test was performed using a modified disk method from the literature [46]. A membrane disk was immersed into a flask containing 5 mL of simulated body fluids (SBFs) (buffer solution) at pH = 7.40 and at 37 °C to determine their biodegradability in human skin. During the experiment, the disintegration time for separate units of membrane disks was observed. In cases where a coherent matrix remained after 168 h, the sample was recorded as not disintegrated. Ten parallels were tested for each formulation (*n* = 10).

#### 3.2.12. Blood Coagulation Time: Prothrombin Time and Activated Partial Thromboplastin Time

The prothrombin time PT was measured to evaluate the effect of the samples on the extrinsic pathway and the activated partial thromboplastin time (APTT) was measured to evaluate the effect of the membrane samples (MControl and MDOX) on the intrinsic pathway [48]. Citrated blood was collected from a healthy adult human donor and the blood was used in the experiments. Then, 60 mg of each membrane was incubated with 600 µL of whole blood in a 2 mL conical-bottom tube. The samples were incubated at 37 °C with oscillatory stirring at 100 rpm for 15 min (MaxQ 4450 Thermo Scientific, Waltham, MA, USA). Immediately after the incubation, the blood from each tube with the membrane sample was collected with a micropipette and then the blood was poured into a 1.5 mL conical-bottom tube; these new samples were centrifuged for 5 min at 3000 rpm to separate the plasma from the blood cells. The plasma was used to measure the PT and the APTT with the Biobas 10 coagulometer (Spinreact México, Naucalpan, Mexico), Estado de Mexico, MEX. Plasma from blood without contact with any sample was used as a physiological control. A normal control and a pathological control (Spinreact México) was used as the negative and positive control, respectively. The blood for these controls was also incubated at 37 °C, at oscillatory stirring (100 rpm, 15 min). The normal PT was set at 11.1–14.3 s according to the reagent instructions. The normal APTT was set at 24–36 s according to the reagent instructions. All experiments were performed by triplicate.

#### 3.2.13. Hemolysis Test

We performed a hemolysis test based on the Standard Practice for Assessment of Hemolytic Properties of Materials (ASTM designation: F 756–00) [49]. The blood from a healthy adult donor was used to assess the hemolytic properties of the experimental membranes. The blood was collected with EDTA vacutainer tubes. Each experimental membrane (60 mg) was incubated with blood (600 µL) in the same way as described in the blood coagulation experiments. Also, blood was collected to separate the plasma from the blood cells in the same way as described for the blood coagulation experiments. The plasma (25 µL) obtained from the blood in contact with the experimental membrane was mixed with a cyanmethemoglobin reagent (225 µL) in a well of a 96-well microplate. Then, the hemoglobin in plasma was measured at 540 nm in a microplate reader (Multiskan FC Thermo Fisher Scientific, Waltham, MA, USA). The hemoglobin concentration was obtained from a calibration curve made with a solution of standard hemoglobin mixed with a cyanmethemoglobin reagent. Hemolysis was expressed as the percentage of hemoglobin released to the total content. Thus, we mixed a sample of blood with cyanmethemoglobin reagent to achieve the 100% of hemolysis. In addition, we used a NaCl solution as a positive control and a silicon sample as a negative control. A physiologic control was performed with plasma collected from blood from the donor. According to the ASTM standard [49], the material might be nonhemolytic (0–2% of hemolysis), or slightly hemolytic (2–5% of hemolysis), or hemolytic (>5% hemolysis). All experiments were performed in triplicate.

### 3.3. Assessing the Antibacterial Activity of CMC/CHS/G/DOX Membranes

The antibacterial capacity of the formulated membranes was evaluated using *S. aureus* and *E.coli* strains, which are commonly associated with the development of foot ulcers in diabetic patients. Additionally, the antimicrobial capacity of the membranes against *S. mutans* was evaluated to explore an alternative use of the membranes in oral cavity diseases, such as caries.

#### Bacterial Growth

*S. mutans* was cultured overnight in a brain heart infusion (BHI) medium, which contains calf brain and bovine heart infusions, peptone, NaCl, glucose, and disodium phosphate. The bacteria were incubated for 24 h at 37 °C under anaerobic conditions with 5% CO_2_, using an ESCO CelCulture CO_2_ incubator, without shaking. After 24 h, the optical density (OD) of the culture was measured at 600 nm using a 100 µL sample. The measured OD value was used to prepare a subculture, adjusting it to an initial OD of 0.05. This step ensured a standardized bacterial concentration for subsequent inhibition tests. *S. aureus* and *E. coli* strains were cultured aerobically in a LB liquid medium at 37 °C with constant shaking (180 rpm). The subculture methodology previously described was applied. Colony-forming units per milliliter (CFU/mL) at an optical density of 0.05 were determined using the Massive Stamping Drop Plate method (MSDP) [50].

To conduct the inhibition tests, CMC/CHS/G/DOX membranes (round, ~5 mm in diameter) were sterilized under UV light for 20 min per side in a class II biosafety cabinet (LABCONCO Purifier^®^ Biological Safety Cabinet, Labconco Corporation, Kansas City, MO, USA). Once sterilized, two membranes were added to the subcultures of each evaluated strain, as well as to BHI or LB liquid medium as controls. The samples were incubated for 24 h under aerobic or anaerobic conditions as previously described. After incubation, the CFU/mL count was determined using the MSDP method on BHI or LB solid medium. For the control samples, 25 µL drops were placed on BHI or LB solid medium and incubated for 24 h under aerobic or anaerobic conditions, depending on the bacterial strain. Additionally, as a secondary control, each membrane was placed directly on BHI or LB solid medium and incubated under the same conditions. This procedure was performed in duplicate, with CFU/mL counts determined in quintuplicate for each repetition. Additionally, the inhibition test was conducted using a technique similar to the sensidisc method, following the methodology described by Hachity-Ortega et al. [10].

### 3.4. Statistical Analysis

All values are presented as the mean ± SD (standard deviation). Statistical analyses were performed using Jamovi 2024, Sydney, AUS [51] to evaluate the data obtained. To compare the water absorbance capacity, swelling index, roughness, moisture sorption, surface pH, tensile strength, elongation, mass and thickness measurements, and antibacterial activity between the two TP, an independent sample *t*-test was employed. The level of significance was set at *p* < 0.05.

## 4. Conclusions

In this study, we successfully synthesized CMC/CHS membranes loaded with doxycycline (DOX) and demonstrated their promising potential as a novel wound delivery system for topical applications, particularly in the treatment of diabetic foot ulcers. The incorporation of DOX into the biopolymeric matrix significantly enhanced the antibacterial properties of the membranes, exhibiting effective inhibition against *S. aureus*, a key pathogen involved in chronic infections associated with diabetic wounds. The membranes also displayed excellent mechanical and physical characteristics, such as appropriate elasticity, moisture retention, and swelling behavior, all of which are crucial for their potential application in wound healing. Additionally, the low hemolytic levels observed in our study indicate that the membranes are biocompatible and exhibit favorable interactions with blood cells, further supporting their suitability for clinical use. Our results demonstrate that the combination of CMC, CHS, and glycerol provides a synergy that improves the mechanical strength and water retention capacity of the membranes, making them ideal for maintaining a moist wound environment, which is a critical factor in promoting wound healing. The findings of this study suggest that biopolymeric membranes offer a viable alternative to traditional wound care treatments. The ease of application, combined with their biocompatibility, drug delivery capability, and antibacterial action, makes these membranes a promising candidate for the treatment of diabetic foot ulcers. However, to fully assess their potential, further kinetic studies and in vivo evaluations are required to explore their effectiveness and long-term performance in clinical settings. These additional investigations will provide critical insights into the ability of these membranes to promote tissue regeneration, reduce infection rates, and improve patient compliance in the management of chronic wounds.

## Figures and Tables

**Table 1:** Overview of characteristics of CMC/CHS/G (MControl) and CMC/CHS/G/DOX (MDOX) membranes, including tensile strength (MPa ± SD) and elongation at break (% ± SD) from stress strain test at 5 mm/min speed (*n* = 10), mass (mg ± SD) (*n* = 10), thickness (µm ± SD) (*n* = 10), surface pH (mean ± SD) at 24 h (*n* = 10), moisture sorption (5) after 10 days (*n* = 10), water sorption at 48 h (% ± SD) (*n* = 10), swelling index (mean ± SD) (*n* = 10), and roughness (Ra and Rz) (*n* = 10) (values are given as mean ±SD) (*n* = 3), blood coagulation time (PT and TT) (values are given as s ± SD) (*n* = 3) and hemolysis (% ± SD) (*n* = 3).

		MControl CMC/CHS/G	MDOX CMC/CHS/G/DOX	*p* Value
Tensile strength (MPa ± SD)		0.09 ± 0.03	0.07 ± 0.02	0.096
Elongation at break (% ± SD)		75.29 ± 2.73	73.52 ± 5.12	0.348
Thickness (µm ± SD)		604.00 ± 182	833.00 ± 134	<0.005
Mass (mg ± SD)		6.05 ± 0.31	9.25 ± 0.69	<0.001
Roughness	Ra (µm ± SD)	0.49 ± 0.25	0.359 ± 0.237	0.241
	Rz (µm ± SD)	3.58 ± 1.658	1.543 ± 1.014	<0.004
Surface pH (mean ± SD)		4.01 ± 0.08	3.87 ± 0.03	<0.002
Moisture sorption (% ± SD)		37.52 ± 6.54	205.01 ± 66.94	<0.002
Water sorption at 48 h (% ± SD)		335.71 ± 128.51	437.81 ± 123.32	0.087
Swelling index (mean ± SD)		0.52 ± 0.05	0.53 ± 0.03	<0.01
Blood coagulation time	PT (s ± SD)	11.50 ± 0.40	11.7 ± 0.70	0.443
	TT (s ± SD)	25.60 ± 3.60	30.1 ± 1.90	<0.002
Hemolysis (% ± SD)		16.80 ± 0.03	14.72 ± 0.05	<0.001

**Table 2:** Inhibition of membranes against the three bacterial species, *S. mutans*, *S. aureus*, and *E. coli*. The average of 10 replicates is shown, 5 replicates in each event.

*Membrane*	*S. mutans*	*S. aureus*	*E. coli*
*Control ^b^*	7.75 ± 0.16	8.35 ± 0.44	8.93 ± 0.68
*MControl*	7.25 mm ± 0.33	8.24 ± 0.23	8.36 ± 0.30
*MDOX*	0.00	0.00	6.21 ± 0.21

^b^ Refers to the number of bacteria without the presence of membranes.

**Table 3:** Sample designation and composition in terms of CMC, CHS, G, and DOX content.

Sample	MControl (%) *	MDOX (%) *
CMC (p/p)	7.56	7.56
CHS (p/p)	3.78	3.78
G (*v*/*v*)	7	7
DOX (p/p)	0	1.08

* Percentage of CMC, CHS and G are based on the solution, and percentage of DOX was based on biopolymers.

## Data Availability

Data is contained within the article. The original contributions present in this study are included in the article. Further inquiries can be directed to the corresponding authors.
